# Antimicrobial proteins and polypeptides in pulmonary innate defence

**DOI:** 10.1186/1465-9921-7-29

**Published:** 2006-02-17

**Authors:** Mark P Rogan, Patrick Geraghty, Catherine M Greene, Shane J O'Neill, Clifford C Taggart, Noel G McElvaney

**Affiliations:** 1 Pulmonary Research Division, Royal College of Surgeons in Ireland, Beaumont Hospital, Dublin, Ireland

## Abstract

Inspired air contains a myriad of potential pathogens, pollutants and inflammatory stimuli. In the normal lung, these pathogens are rarely problematic. This is because the epithelial lining fluid in the lung is rich in many innate immunity proteins and peptides that provide a powerful anti-microbial screen. These defensive proteins have anti-bacterial, anti- viral and in some cases, even anti-fungal properties. Their antimicrobial effects are as diverse as inhibition of biofilm formation and prevention of viral replication. The innate immunity proteins and peptides also play key immunomodulatory roles. They are involved in many key processes such as opsonisation facilitating phagocytosis of bacteria and viruses by macrophages and monocytes. They act as important mediators in inflammatory pathways and are capable of binding bacterial endotoxins and CPG motifs. They can also influence expression of adhesion molecules as well as acting as powerful anti-oxidants and anti-proteases. Exciting new antimicrobial and immunomodulatory functions are being elucidated for existing proteins that were previously thought to be of lesser importance. The potential therapeutic applications of these proteins and peptides in combating infection and preventing inflammation are the subject of ongoing research that holds much promise for the future.

## Introduction

The host response to bacterial infection of the airways is dependent on both innate (non-antibody-mediated) and adaptive (antibody-mediated) immune systems. The acquired immune system is primarily cellular in composition relying on the actions of B and T cells that are prolonged in activation and duration. However, the innate immune response is more immediate and depends on the activity of phagocytic cells such as macrophages and neutrophils and in the expression of a number of proteins and peptides, some of which are secreted by the respiratory tract epithelium and phagocytic cells. The rapidity of the innate immune system provides effective host defense against a vast array of microbes in a manner that is independent of prior exposure to the invading pathogen [1].

Unlike any of the other vital organs, the lung is exposed daily to a large amount of pathogens present in air and is potentially vulnerable to infection and inflammation. For optimal gas exchange, the lung has a vast surface area (150 m^2^), a very thin delicate epithelium and extensive blood flow. Inherent in this structure is an enormous immunological burden. The 11,000–15,000 liters of air inhaled daily contain a myriad of pathogens, pollutants and allergens. In the normal lung, many inhaled microbes are trapped in the mucus layer coating the nasal epithelium and upper respiratory tract. Once trapped, they can be transported by ciliary motion to the pharynx and swallowed. For organisms that evade mucociliary clearance, further protective immune mechanisms act locally to facilitate clearance of inhaled pathogens and to modulate inflammatory responses.

Organisms that reach the alveolar compartment are deposited in the epithelial lining fluid (ELF), a thin aqueous film containing pulmonary surfactant that lines the gas-exchanging surface of the pulmonary epithelium. Whenever this deposition occurs, the invader and the host initiate a series of complex offensive and defensive strategies. Sensing of the physiologic body temperature and the pH and ionic strength of the epithelial lining fluid (ELF) by the organism triggers a program of gene expression designed to optimize survival under adverse conditions. These include up regulation of microbial genes required for proliferation and host evasion and down regulation of genes that regulate less necessary functions. The lung response to this threat is coordinated by the pulmonary epithelium and alveolar macrophages, which release cytokines and chemokines to recruit additional inflammatory cells to the airspace.

In the upper respiratory tract, nasal, tracheal, and bronchial secretions are generated by airway epithelial cells, especially the goblet cells; by the sub mucosal glands; by transudation and transport of proteins from plasma; and by resident and recruited phagocytes, neutrophils, eosinophils, monocytes, and macrophages. Distally the airways are lined with ELF, which is composed of airway and alveolar secretions. The composition of ELF varies by anatomical location and in response to mechanical, chemical, and microbial stimulation. In the distal airways and alveoli, Clara cells and type 2 alveolar cells are, respectively, the predominant secretory epithelial cells. For the naive host, the primary antimicrobial defences in ELF are the resident alveolar macrophages and protein components of the innate immune system. These intrinsic antimicrobial properties of respiratory secretions act in concert with the mechanical and phagocytic clearance mechanisms to defend the respiratory tract against colonization or invasion by environmental microbes.

As first described by Alexander Fleming over 80 years ago (Fleming, 1922), upper airway secretions contain lysozyme which possesses intrinsic microbicidal and bacteriostatic properties. Since these early observations, a diverse range of antimicrobial proteins and polypeptides that are broadly antimicrobial and predominantly cationic have been characterised and elucidated in respiratory secretions. The aim of this review is to highlight the many rolls of the various antimicrobial proteins and polypeptides in pulmonary innate defense.

ELF is rich in innate immunity proteins including the cell wall-degrading enzyme lysozyme, the iron-chelating protein lactoferrin, the anti-elastase seceretory leucoprotease inhibitor and specific membrane-permeabilizing members of the defensin, cathelicidin, and pentraxin families. There are a myriad of other such proteins including bactericidal permeability increasing protein (BPI), surfactant proteins A-D and other collectins. New innate immunity proteins are being discovered frequently and new, previously unknown defensive and immunomodulatory properties are being elucidated in existing proteins that were previously thought to be unimportant. In the normal lung, such proteins ensure that the process of pathogen elimination works smoothly and seamlessly in most cases. Antimicrobial peptides, defensins and cathelicidins, have also been discovered which have a wide range of microbicidal activities against Gram-positive and Gram-negative bacteria. These peptides share some common features including a large number of positively charged residues and the ability to assume amphiphilic conformations such as α-helices or β-sheets. The most abundant airway antimicrobial factors are lysozyme, lactoferrin, secretory leucoprotease inhibitor (SLPI), human beta defensin peptides and the cathelicidin, LL-37 respectively [2]. Bactericidal Permeability Increasing Protein (BPI) and the collectins, surfactant proteins A and D also play key antimicrobial and immunomodulatory roles. A summary of the key innate immunity proteins is shown in Table 1.

**Table 1:** Structure, anti-microbial and immunomodulatory activities of innate immunity proteins and peptides

**Protein**	**Structure**	**pI**	**Pulmonary Source**	**Antimicrobial activity**	**Immunomodulatory activity**
Lactoferrin	Iron- binding glycoprotein MW 80 kDa	8.7	Neutrophil secondary specific granules and submucosal gland epithelial cells	Bactericidal Bacteriostatic Inhibits biofilm formation Anti-fungal Anti-viral	Binds LPS and can prevent septic shock. Binds CPG motifs. Anti-oxidant.
SLPI	11.7 kDA non-glcosylated protein	>9.2	Macrophages, epithelial cells, neutrophils	Bactericidal Bacteriostatic Anti-viral	Powerful anti-protease Inhibits LPS-induced NF-κB activation. Attenuates pulmonary recruitment of neutrophils in sepsis models. Impairs LTA and LPS induced proinflammatory gene expression in monocytes and macrophages.
Lysozyme	14 kDA enzyme	10.5	Neutrophil secondary specific granules and submucosal gland epithelial cells	Bactericidal Bacteriostatic	Unknown
Human Defensins	3–5 kDA peptides	8.8–9.5	Neutrophil azurophil granules and pulmonary epithelial cells	Bactericidal Bacteriostatic Anti-parasitic Anti-fungal Anti-viral	Mitogenic and chemotactic activities. Contribute to epithelial repair in the lung by enhancing epithelial cell proliferation.
LL-37	Peptide that requires proteolytic processing to liberate the mature functional antimicrobial peptide.		Neutrophil secondary specific granules and submucosal gland epithelial cells	Bactericidal Bacteriostatic Anti-fungal Anti-viral	Reduced production of the pro-inflammatory cytokine TNF-α from macrophages stimulated with LPS and may be responsible for the migration of immune cells to areas of inflammation and infection
BPI	55-kDa protein	9.6	Neutrophil primary granules	Bactericidal Bacteriostatic Act as an opsonin to enhance neutrophil phagocytosis.	Downregulates LPS and other endotoxins *in vivo*
Surfactant proteins A and D	Lipoprotein complex	4.5–5.4	Type II pneumocytes, airway Clara cells	Key opsonins facilitating phagocytosis of bactaeria and viruses. Bactericidal Bacteriostatic Anti-fungal Anti-viral	Both SP-A and SP-D have the capacity to modulate multiple leucocyte functions
Lactoperoxidase	Enzyme		Airway epithelium	Bactericidal Anti-fungal Anti-viral	
CCL20	Chemokine with similar structure to HBD		Airway epithelium	Bactericidal against Gram negative bacteria	Stimulates the migration of B-cells, immature dendritic cells, and a subset of memory T cells

## Expression of innate immunity proteins

Expression of many antimicrobial polypeptides is modulated locally by inflammation. Inflammatory stimuli release chemoattractants that recruit neutrophils containing large amounts of antimicrobial proteins/peptides and a number of hydrolytic enzymes. Alpha defensins, lysozyme (both primary and secondary granules) and bacteria permeability increasing protein (BPI) are all contained in neutrophil primary granules from where they can be degranulated into the phagosome thereby exposing ingested microorganisms to high concentrations of granule contents. Secondary granules contain distinct antimicrobial proteins and peptides (e.g lactoferrin) which are deployed toward the leading edge of the chemotaxing neutrophil from where they are readily and rapidly degranulated extracellularly [3].

Furthermore, granular contents of neutrophils can be released into inflammatory fluids following neutrophil death, so called "holocrine secretion" [4]. Neutrophils engulf and kill bacteria when their antimicrobial granules fuse with the phagosome. Zychlinsky et al have recently described an additional neutrophil killing technique: Activated neutrophils release granule proteins and chromatin that together form extracellular fibers that bind Gram-positive and -negative bacteria. These neutrophil extracellular traps (NETs) degrade virulence factors and kill bacteria. NETs appear to be a form of innate response that binds microorganisms, prevents them from spreading, and ensures a high local concentration of antimicrobial agents to degrade virulence factors and kill bacteria [5]. In addition to triggering release of antimicrobials from circulating blood cells, inflammatory stimuli may also increase the synthesis of innate immunity proteins such as β-defensin by epithelial cells [6,7] and, when chronic, induce the differentiation of respiratory epithelial cells into secretory cell types. Lung epithelial cells and submucosal glands have been show to express other key innate immunity proteins including lactoferrin and LL-37 [8,9]. Lysozyme has been demonstrated in secretory granules of serous but not mucous cells in airway submucosal glands, and was absent from the surface epithelium, cartilage, and connective tissue [10,11]. We shall look at some of these defensive proteins and peptides in greater detail.

### (i) Lactoferrin

Lactoferrin is an iron-binding glycoprotein (Mw 80-kDa) and as such it exists in both iron-replete and iron-depleted forms. The iron-depleted form of lactoferrin is its more biologically active form. Lactoferrin is a cationic protein with an isoelectric point (pI) of 8.7 and is widespread in human secretions. It is found in high concentrations in breast milk (~3–7 mg/ml), tear fluid (1–4 mg/ml), vaginal secretions, gut-lining fluid, cervical mucus plugs, saliva, exocrine secretions and respiratory secretions (0.1–1 mg/ml). Lactoferrin is released from neutrophil secondary (specific) granules at areas of inflammation. Like many innate immunity proteins, lactoferrin is highly cationic (calculated pI = 8.5). Lactoferrin is both antimicrobial and anti-inflammatory and contributes to host defense both systemically and at mucosal surfaces. Its antimicrobial effects include being directly bactericidal and bacteriostatic and more recently it has been shown to inhibit *Pseudomonas *biofilm formation by a separate mechanism [12]. Lactoferrin exhibits antibacterial effects on Gram-negative bacteria by means of two mechanisms. Firstly, by binding iron, it limits the amount of free iron (an essential growth factor for microorganisms) available. For example, in one study *Streptococcus mutans *and *Vibrio cholerae *were killed by incubation with purified human apolactoferrin, the iron-depleted form of the protein. Concentrations of lactoferrin below that necessary for total inhibition resulted in a marked reduction in viable colony-forming units. This bactericidal effect was contingent upon the metal-chelating properties of the lactoferrin molecule [13]. Lactoferrin contains two high-affinity ferric iron binding sites facilitating such iron sequestration from pathogenic microbes. Secondly, lactoferrin can destabilise the outer membrane of gram-negative bacteria by binding to it resulting in altered permeability that leads to microbial injury and death. This activity has been attributed to the 17 amino acid N-terminal portion of lactoferrin. This portion is rich in arginine residues that give the molecule its highly cationic nature and has been termed lactoferricin. The related iron-binding protein transferrin lacks these arginine residues and is less cationic (pI of 5–5.5) [14]. Lactoferrin has been shown to kill clinical strains of *E.coli, S. aureus *and mucoid *P. aeruginosa *isolated from CF airways [15]. Lactoferrin acts synergistically with other innate immunity proteins such as lysozyme and SLPI in bacterial killing [16,17]. It has also been shown that lactoferrin enhances the antimicrobial effects of some antibiotics [18]. Lactoferrin reduces the minimum inhibitory concentration (MIC) and the minimum bactericidal concentration (MBC) of doxycycline for *Burkholderia cepacia *and *P. aeruginosa *strains with MICs for *B. cepacia *falling from highly resistant to clinically achievable levels [19]. By binding iron, lactoferrin can also act as an anti-oxidant since iron bound to the protein is unable to participate as a catalyst for the generation of free hydroxyl radicals via the Haber-Weiss reaction [20-22].

Lactoferrin has recently been shown to inhibit *Pseudomonas *biofilm formation [12]. The inhibition of biofilm formation is a property unique to lactoferrin and thus it plays a pivotal role in host defense against this highly destructive mode of bacterial growth. Biofilm bacteria are notoriously resistant to host killing and antibiotics. They may be up to one thousand times more resistant to antibiotics than their free-swimming, planktonic counterparts [23,24]. In the presence of lactoferrin, free iron levels decreased inducing a twitching motion in bacteria. This twitching ensured that the bacteria wandered across a surface rather than stopping to form microcolonies, clusters and subsequent pillar and mushroom-shaped mature biofilms. Furthermore, Singh et al demonstrated increased susceptibility of biofilm bacteria to tobramycin in the presence of lactoferrin. We have shown previously that lactoferrin levels are depleted in BAL from Cystic Fibrosis patients with active *Pseudomonas *infections compared to those with no active *Pseudomonas *infection and that this depletion results in impaired ability to prevent *Pseudomonas *biofilm formation [25].

Lactoferrin also has been shown to be active against a number of viruses including human immunodeficiency virus (HIV) and cytomegalovirus (CMV) [26,27]. Lactoferrin is known to prevent replication of hepatitis B and C viruses and is being investigated clinically together with interferon as a potential therapeutic agent in these conditions [28,29]. A recent study elucidated the potential of oral administration of lactoferrin to attenuate pneumonia in influenza-virus-infected mice through the suppression of infiltration of inflammatory cells in the lung [30]. In addition to antiviral properties, lactoferrin also possesses anti-fungal properties: it has been shown to be active against *Candida *species and is being investigated as a potential treatment for oral candidiasis [31].

**Figure 1:**
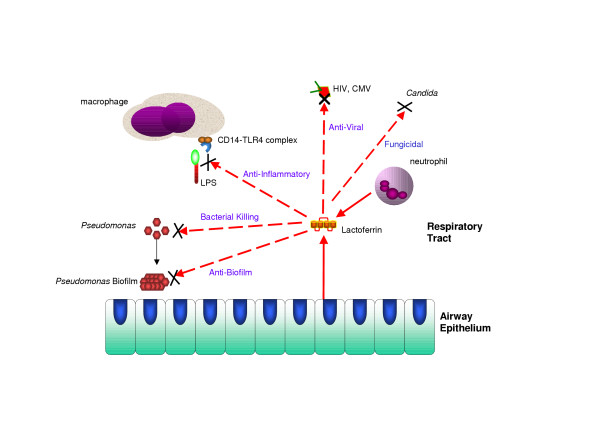
**Multifunctional properties of lactoferrin**. Lactoferrin is released from neutrophils and respiratory tract epithelium and has multiple activities including anti-inflammatory, anti-viral, anti-lipopolysaccharide, anti-biofilm, antibacterial and anti-fungal properties.

Lactoferrin is also a key anti-inflammatory protein. It possesses two basic cradles at residues 1 to 5 and 28 to 34 of the N-terminal end that can bind anionic molecules such as lipopolysaccharide (LPS), heparin and heparin sulfates. Lactoferrin has been shown to inhibit the LPS-induced expression and proteoglycan binding ability of Interleukin-8 in human endothelial cells [32]. Lactoferrin protects against sublethal doses of LPS in mice and germfree piglets. Animals that received pre-treatment with lactoferrin showed minimal effects when given an intra-peritoneal injection of LPS whereas the control group of animals that did not receive pre-treatment with lactoferrin succumbed rapidly to septic shock [33]. Lactoferrin has also been shown to lower the expression of adhesion molecules E-selectin and ICAM-1 on endothelial cells further modifying the immune response [34]. Another immunomodulatory function of lactoferrin stems from its ability to bind unmethylated CpG motifs, which are bacterial DNA products capable of stimulating various innate and acquired immune responses in human and murine models [20]. A schematic of lactoferrin's many antimicrobial and immunomodulatory roles is shown in figure 1.

### (ii) Secretory Leucoprotease Inhibitor (SLPI)

SLPI is a 11.7-kDa non-glycosylated protein that is expressed by macrophages, neutrophils and the mucosal surface of epithelial cells [35,36]. It is a highly basic (pI > 9.5), acid-stable but alkaline-labile protein [37]. SLPI is the third most abundant innate immunity protein of respiratory secretions after lysozyme and lactoferrin. It is estimated that SLPI is present at concentrations of 0.1 to 2 ug/ml in airway lavage fluid [38,39] and 2.5 ug/ml in nasal secretions[40]. SLPI through its C terminal domain is a serine protease inhibitor and provides significant protection against neutrophil elastase, a powerful elastolytic enzyme released from neutrophils during degranulation at areas of infection and inflammation. SLPI also inhibits the serine protease cathepsin G as part of its anti-protease effects in the lung [41]. In addition to its potent anti-protease activity, SLPI has important anti-bacterial, anti-viral and anti-inflammatory properties. The N-terminal domain of SLPI has modest antimicrobial activity *in vitro *against Gram-negative and Gram-positive bacteria [42]. SLPI has been shown to inhibit human immunodeficiency virus (HIV) infectivity of monocytes by blocking viral DNA synthesis. Saliva is rich in SLPI and it is felt that this accounts for the low viral transmission rates via saliva [43,44]. Prior administration of SLPI to rats attenuated pulmonary recruitment of neutrophils in an immunoglobulin G (IgG) immune complex model of acute lung injury [45]. Pre-treatment with SLPI greatly reduced inflammation in both liver and lungs in a mouse model of hepatic ischaemia/reperfusion injury [46]. SLPI can inhibit LPS-induced NF-κB activation by inhibiting degradation of IRAK, IκBα and IκBβ [47] and can also impair lipoteichoic acid (LTA) and LPS induced pro-inflammatory gene expression in monocytes and macrophages in vitro [36,48]. In addition to its antiprotease activity, SLPI has been shown to exhibit anti-inflammatory properties, including down-regulation of tumor necrosis factor alpha expression by lipopolysaccharide (LPS) in macrophages and inhibition of nuclear factor (NF)-kappaB activation in a rat model of acute lung injury. SLPI has recently been shown to enter cells, becoming rapidly localized to the cytoplasm and nucleus where it affects NF-kappaB activation by binding directly to NF-kappaB binding sites in a site-specific manner [47]. However once oxidised, the anti-inflammatory and anti-elastase effects of SLPI are diminished. Greene et al. demonstrated cleavage of SLPI in infected lobes in community acquire pneumonia resulting in impaired anti-NE activity. SLPI was inactivated by cleavage, oxidation and complex formation paving the way for free neutrophil elastase to exacerbate pulmonary parenchymal inflammation and tissue damage [49]. SLPI is susceptible to protease cleavage. Cathepsins B, L, and S have been shown to cleave and inactivate SLPI. Analysis of epithelial lining fluid samples from individuals with emphysema indicated the presence of active cathepsin L and cleaved SLPI [50]. Serine and cysteine proteases produced by the house dust mite in asthma have been shown to cleave SLPI and may increase the susceptibility of patients with allergic inflammation to infection [36].

### (iii) Lysozyme

Lysozyme is a 14-kDa enzyme directed against the β 1→4 glycosidic bond between *N*-acetylglucosamine and *N*-acetylmuramic acid residues that make up peptidoglycan, the cell wall material that gives bacteria their shape. Lysozyme is a basic protein with a pI of 10.5 [51]. It is stored in both primary and secondary neutrophil granules. In addition to enzymatic lysis of bacterial cell walls, lysozyme can also kill bacteria by a non-enzymatic mechanism [52]. Lysozyme is highly active against many Gram-positive species, including *Bacillus megaterium*, *Micrococcus luteus*, and many streptococci. Lysozyme also has an important role against Gram-negative organisms. Its ability to kill Gram-negative organisms may be influenced by ionic concentration, osmolarity, and the presence of synergistic cofactors [2,15,17].

As lactoferrin and lysozyme are present together in high levels in mucosal secretions and neutrophil granules, it is probable that their interaction contributes to host defense [17]. Lactoferrin in concert with other cofactors presumably disrupt the outer membrane of Gram-negative bacteria and allow lysozyme access to the sensitive peptidoglycan layer. Lysozyme is a component of both phagocytic and secretory granules of neutrophils and is also produced by monocytes, macrophages, and epithelial cells. Both lysozyme and lactoferrin arise in the lower respiratory tract within the airways and their levels are elevated in association with chronic bronchitis suggesting that lactoferrin and lysozyme may contribute to the modulation of airway inflammation in chronic bronchitis. Lysozyme is about tenfold more abundant in the initial "airway" aliquot than in subsequent aliquots of bronchoalveolar lavage [53], and its concentration correlates poorly with neutrophil concentrations, suggesting that, in general, airway epithelium and its glands are the major sources of lysozyme in airway secretions. Lysozyme is present at concentrations of between 0.1 and 1 mg/ml in respiratory secretions. In one study to assess the role of lysozyme in pulmonary host defense *in vivo*, transgenic mice expressing rat lysozyme cDNA in distal respiratory epithelial cells were generated. Two transgenic mouse lines were established in which the level of lysozyme protein in bronchoalveolar lavage (BAL) fluid was increased 2- or 4-fold relative to that in wild type (WT) mice. Lysozyme activity in BAL was significantly increased (6.6- and 17-fold) in 5-wk-old animals from each transgenic line. Killing of group B *streptococc*i was significantly enhanced (2- and 3-fold) in the mouse transgenic lines at 6 h following infection and was accompanied by a decrease in systemic dissemination of pathogen. Killing of *Pseudomonas aeruginosa *was also enhanced in the transgenic lines (5- and 30-fold). Twenty-four hours after administration of *Pseudomonas aeruginosa*, all transgenic mice survived, whereas 20% of the WT mice died. The authors concluded that increased production of lysozyme in respiratory epithelial cells of transgenic mice enhanced bacterial killing in the lung *in vivo*, and was associated with decreased systemic dissemination of pathogen and increased survival following infection [54]. A recent *in vivo *study of lysozyme derived from submucosal glands in ferret trachea demonstrated that lysozyme-depleted secretions were much less effective at inhibiting bacterial growth than mock-depleted samples, suggesting that lysozyme is partially responsible for the antibacterial activity of the glandular airway secretions. Furthermore, depletion of lysozyme from human nasal secretions also reduced antibacterial activity by 50% [55].

### (iv) Defensins

Defensins are 3- to 5-kDa peptide members of a widely distributed family with characteristic three-dimensional folding with six cysteine-three disulfide patterns. Defensins have broad cytotoxic activity against bacteria, fungi, parasites, viruses and even host cells [56]. Defensins are subdivided into different classes that include alpha and beta defensins. Alpha defensins are also known as human neutrophil peptides (HNPs). HNP-1 to -4 are found in azurophil granules of neutrophils where they constitute up to 50% of the total protein present [57]. HNP-5 and -6 have been identified in Paneth cells in the crypts of the small intestinal mucosa and also in the female reproductive tract. The alpha defensins have a wide variety of actions including mitogenic and chemotactic activities [58,59]. Alpha defensins contribute to epithelial repair in the lung by enhancing epithelial cell proliferation [60]. The more recently identified human beta defensins (HBDs) 1–4 differ slightly from classical alpha defensins in the spacing and connectivity of their cysteines [2]. Beta defensins have five to eight positively charged residues resulting in quite similar (calculated) isoelectric points of 8.9 to 9.5 [61]. HBD-2 and -3 are secreted in response to LPS and cytokines (TNFα, interleukin-1 beta) and are active against Gram-positive (HBD-3) and Gram-negative (HBD-1, -2 and -3) bacteria whilst HBD-1 is upregulated by interferon-gamma (IFN-γ) [62-64]. We have previously demonstrated that human lung epithelial cells express Toll-like receptor four (TLR4) on their surface and that stimulation of these cells with *Pseudomonas *LPS results in increased HBD2 gene and protein expression [65]. All three beta defensins have been identified in lung and they have been shown to act synergistically with other innate immunity proteins in bacterial killing [15]. Individual beta defensins have differential antimicrobial activity. *Staphylococcus aureus *is resistant to killing by HBD-1 and HBD-2 but even strains of this organism that are multi-drug resistant are susceptible and sensitive to killing by HBD-3 [66]. We have shown previously that HBD 2 and 3 are susceptible to proteolytic cleavage by cysteinyl cathepsins that are present at elevated concentrations in the Cystic Fibrosis and COPD airways [49]. New families of defensins and individual defensins are being discovered. A novel family of antimicrobial peptides termed "theta defensins" has been described in monocytes and macrophages of macaque monkeys. Theta defensins are naturally produced by a unique ligation of two truncated alpha defensins [67]. Whilst they are important in their own right, β-defensins are present at much lower concentrations than lysozyme, SLPI or lactoferrin and thus their overall contribution to host defense must be taken in context [15].

### (v) Cathelicidin LL 37

Cathelicidins are a family of antimicrobial proteins found in neutrophil specific granules of which LL-37 is the only human member identified to date [9,68,69]. LL-37 is also present in certain lymphocytes, testicular tissue and airway epithelium [9,68]. Cathelicidins are stored as inactive pro-peptides precursors and require processing to become active peptides [70]. LL-37 is activated when proteinase 3 cleaves its precursor, hCAP-18. Some cathelicidin genes possess upstream DNA motifs (eg. NF-κB) predicted to convey inducibility during acute phase responses [71]. LL-37 has been shown to reduce bacterial load by *Pseudomonas *when over-expressed in murine models [72]. Furthermore it conveys improved survival following administration of lethal doses of LPS [72]. LL-37 also reduced production of the pro-inflammatory cytokine TNF-α from macrophages stimulated with LPS and may be responsible for the migration of immune cells to areas of inflammation and infection [73]. Protegrin, the porcine equivalent of LL-37 is currently being evaluated as an anti-inflammatory/antimicrobial agent in patients with mucositis post chemotherapy [3]. LL-37 activity may be impaired in Cystic Fibrosis where anionic filaments of F-actin and DNA bind to it and inhibit its bactericidal action. Addition of the actin filament-fragmenting protein gelsolin frees LL-37 from this binding and restores antimicrobial activity [72].

### (vi) Bactericidal Permeability-Increasing protein (BPI)

BPI is a 55-kDa protein that is predominantly active against Gram-negative bacteria [2,74]. It is stored in neutrophil primary granules and exerts its effects through three distinct mechanisms: firstly, it is directly cytotoxic via its effects on bacterial membranes; secondly, it acts as an opsonin to enhance neutrophil phagocytosis and thirdly, it can neutralise bacterial LPS [75]. In common with many innate immunity proteins, BPI possesses a highly cationic N-terminal end which contains its bactericidal and endotoxin neutralising zones [75,76]. As with many of these defensive proteins, BPI acts synergistically with other members of the innate immune system such as cathelicidins and defensins in bacterial killing. It also acts in concert with the complement system [76]. BPI is thought to have a role in down-regulating the pro-inflammatory effects of gram-negative bacteria and endotoxins *in vivo *[77].

### (vii) Collectins - Surfactant Proteins A and D

Pulmonary surfactant is a lipoprotein complex that is synthesised by type II pneumocytes and by airway Clara cells. It is secreted into the epithelial lining fluid where it modulates surface tension. More recently surfactant has been shown to play a role in host defense against infection and inflammation. Surfactant proteins belong to the collagen-like-lectin or collectin family that also includes mannose binding protein bovine coglutinin and CL-43 [78,79]. Collectins share an N-terminal collagen-like domain and a C-terminal lectin or Carbohydrate recognition domain (CRD) domain capable of binding carbohydrates in a calcium-dependent manner. These C-type lectin domains can bind oligosaccharides found on bacterial, non-encapsulated fungal as well as viral envelope surfaces [79]. Surfactant consists of the four surfactant proteins 1–4 bound to phospholipids and is responsible for reducing surface tension at the air liquid interface within the alveoli in lung. Surfactant proteins are key opsonins facilitating phagocytosis of bacteria and viruses by macrophages and monocytes [80] Both SP-A and SP-D have been shown to be directly bactericidal against *E.coli*, while SP-A and SP-D are fungicidal against *Histoplasma Capsulatum*. SP-A and D deficient mice were unable to kill this fungus [81]. Both SP-A and SP-D have the capacity to modulate multiple leucocyte functions [78,82]. The addition of SP-A to cultures of *Mycoplasma. pneumoniae *markedly attenuated the growth of the organism assessed by colony formation, metabolic activity, and DNA replication. The bacteriostatic effects of SP-A were reversed by dipalmitoylphosphatidylglycerol. These findings demonstrate that human SP-A can play a direct role in antibody-independent immunity to *M. pneumoniae *by interacting with lipid ligands expressed on the surface of the organism and implicate SP-A in the immediate host response to the bacteria [83].

Recent research has focused on the proteolytic cleavage of SP-A and D by various proteases in the lung. Proteolytic damage to surfactant protein by neutrophil elastase and cathepsin G was demonstrated in bronchoalveolar lavage fluid of cystic fibrosis patients [84]. The bacterial protease, *Pseudomonas aeruginosa *elastase was shown to degrade SP-A and SP-D [85]. Furthermore, cleavage of SP-D by this enzyme results in failure of the surfactant to bind or aggregate bacteria that are aggregated by intact SP-D. Thus, cleavage eliminates many of SP-D's normal immune functions [86].

### (viii) Lactoperoxidase

Early animal studies showed airway mucosa secretes an enzyme known as peroxidase that was active in preventing infection of the airway[87]. Gerson et al subsequently demonstrated production of the biocidal compound hypothiocyanite *in vitro *by airway lactoperoxidase (LPO). They also showed *in vivo *inhibition of airway LPO in sheep leads to a significant decrease in bacterial clearance from the airways. Their data suggest that the LPO system is a major contributor to airway defenses [88]. The airway LPO system may provide additional protection against viral [89-91] and fungal infections [92,93]. LPO was subsequently demonstrated in human airways airway secretions with activity against *Pseudomonas aeruginosa, Burkholderia cepacia *and *Haemophilus influenzae *[94].

### (ix) CCL20

Chemokine ligand 20 (CCL20) shares similar structural and functional properties with human beta-defensins (HBDs) Airway epithelial cells have been shown to express CCL20 [95]. The inflammatory cytokines interleukin (IL)-1β and tumor necrosis factor-α (TNF-α) upregulate expression of this protein via the nuclear factor (NF)-κB pathway [96,97]. CCL20 is also produced by neutrophils [98]. It has been shown to exert antimicrobial activity against a wide spectrum of mainly Gram-negative bacteria [99]. Starner et al demonstrated that CF BAL contains elevated concentrations of CCL20 compared to normal BAL and that CCL20 exhibited salt-sensitive bactericidal activity [95].

## Potential therapeutic applications

The use of antimicrobial peptides and proteins as potential therapeutic targets is an attractive concept. Theoretically, such compounds would have low immunogenicity and high bioavailability with minimal toxicity [2]. The costs involved in developing, producing and administering such compounds should be outweighed by the enormous potential benefits in an era where antibiotic resistant has reached crisis proportions. Gene therapy as a means of augmenting levels of active antimicrobial proteins (cathelicidins) was previously investigated [72] although there has been little progression in developing this early work. Alternatively, innate immunity proteins such as lactoferrin could potentially be aerosolized directly into the lungs in CF as is currently being evaluated with protegrin, a porcine-derived cathelicidin [3]. Recombinant human lactoferrin (rHLF) is relatively cheap to manufacture and of low antigenic potential. Inhaled rHLF has been shown to reduce the late phase response to antigenic stimuli in sheep [100]. It has been shown to be safe in a phase II clinical trial based on its immunomodulatory effects in asthma in humans [101]. Another strategy could involve protecting native antimicrobials from proteolytic degradation by proteases. Surfactant proteins are susceptible to protease cleavage by bacteria-derived proteases [86]. We have shown previously that lactoferrin [25], SLPI [50] and human β-Defensins [49] are all rapidly degraded by cysteinyl cathepsins that are over-expressed in chronic lung diseaes such as Cystic Fibrosis and COPD. Cleavage of innate immunity proteins results in loss of their antimicrobial effects. Cystatins are naturally occurring cathepsin inhibitors that appear to be overwhelmed by the sheer cathepsin burden in these conditions. Design of a selective pharmaceutical cathepsin inhibitor based on cystatins or small synthetic cathepsin inhibitors could minimize cleavage of these innate immunity proteins, thereby optimizing the pulmonary antimicrobial screen. Perhaps the ideal combination would be a nebulised innate immunity protein (eg lactoferrin) to augment depleted natural levels coupled with a synthetic cathepsin inhibitor to prevent proteolytic degradation. Implementation of such a regime early in CF before biofilms have taken hold could potentially reduce morbidity and mortality from *P. aeruginosa *infections in CF significantly. Strategies to inhibit innate immunity protein cleavage by other proteases such as NE and bacterial proteases may also have therapeutic potential. The emergence of widespread resistance to many conventional antibiotics and the selecting out of multi-drug resistant "super-bugs" should prompt further investigation into potential roles for innate immunity proteins in the clinical arena. Synergistic combinations of innate immunity proteins with existing antibacterial and antifungal agents should continue to be evaluated [102].

## Conclusion

Microbes are capable of rapid adaptation to changing environmental conditions to maximise survival and increase pathogenicity. Species diversity and genetic heterogeneity lead to multiple virulence factors among microorganisms in the respiratory tract. In order to combat this threat, the lung is endowed with incredibly powerful and potent antimicrobial and anti-inflammatory proteins that also have rolls in epithelial repair. Despite recent advances in our knowledge of the complex roles and functions of the various innate immunity proteins in pulmonary infection and inflammation, further research is needed to characterize and elucidate specific biological functions and pathways of individual proteins. Strategies to augment innate immunity proteins or to prevent their degradation may provide future therapeutic options. As our understanding of this key area grows, we must learn to harness these "natural born killers" and derive maximum clinical benefit from them.

## Competing interests

The author(s) declare that they have no competing interests.

## Authors' contributions

MR and CT drafted the manuscript. All authors read and approved the final manuscript
